# An Economic Comparison in the Elderly of Adjuvanted Quadrivalent Influenza Vaccine with Recombinant Quadrivalent Influenza Vaccine in Spain

**DOI:** 10.3390/vaccines11020427

**Published:** 2023-02-13

**Authors:** Jesús Ruiz-Aragón, Sergio Márquez-Peláez

**Affiliations:** 1 Hospital de La Línea, 11300 La Línea de la Concepción, Spain; 2 Department of Economics, Faculty of Business, Universidad Pablo de Olavide, 41013 Sevilla, Spain

**Keywords:** influenza, vaccination, Spain, cost effectiveness, adjuvanted, recombinant, burden of illness

## Abstract

Influenza infections impose a high burden of morbidity and mortality among older adults, at great cost to individuals and society. Enhanced influenza vaccines, which contain either an immune adjuvant or higher than normal doses of influenza virus antigens, are recommended for older adults. We used a health economics model to evaluate the cost effectiveness in Spain of a recently licensed recombinant quadrivalent influenza vaccine (QIVr), which contains three times the standard dose of influenza virus hemagglutinin but no neuraminidase, compared with an MF59-adjuvanted quadrivalent influenza vaccine (aQIV). Based on current tender prices in Spain and a conservative assumption that QIVr is 10.7% relatively more effective than aQIV, the incremental cost-effectiveness ratio (ICER) for QIVr was EUR 101,612.41 per quality-adjusted life year (QALY) gained. To meet the Spanish willingness-to-pay threshold of EUR 25,000 per QALY gained, QIVr would need to be 34.1% relatively more effective than aQIV. In a probabilistic sensitivity analysis conducted to confirm the robustness of the analysis, 99.7% of simulations for QIVr were higher than the willingness-to-pay curve. These findings suggest that QIVr is not currently a cost-effective influenza vaccine option relative to aQIV for older persons living in Spain.

## 1. Introduction

Seasonal influenza carries a high burden of disease on individuals and society. Of ~1 billion infections occurring worldwide each year, 3–5 million are severe and up to 650,000 result in death—67% of which occur among persons aged 65 years and older [1,2]. The morbidity and mortality burden extends beyond older individuals. During the 2019–2020 season in Spain, influenza infections among persons ≥65 years of age accounted for 26% of all hospitalizations, 40% of all intensive care unit (ICU) admissions, and 37% of all deaths in the country [3]. Among those infected with influenza in Spain between 2010 and 2016, young children aged <4 years had the highest rates of severe cases (18.9 per 100,000) and ICU admissions (4.9 per 100,000), but the rates for adults ≥65 years of age were only slightly lower (16.5 and 4.5 per 100,000, respectively), and older people had by far the highest mortality (3.0 per 100,000, with all other age groups <1 per 100,000) [4]. The direct and indirect costs associated with medical care and work absences in Spain amount to EUR 1 billion per year [5]. A notable portion of these indirect costs are due to family members or other close associates missing work to care the sick, whether children or elderly.

Older adults are not only more vulnerable to influenza complications and death but also may be more susceptible to influenza infection due to immunosenescence, an age-related process that alters the immune system so it responds less effectively to infections or vaccines. In older persons, a systemic low-grade inflammatory state leads to the dysregulation of the innate and adaptive immune systems, and humoral and cellular immune responses against pathogens or in response to vaccines are rendered less effective [6]. For these reasons, annual influenza vaccination is recommended for older adults in many countries, including Spain [7]. Enhanced vaccines were specifically developed to overcome immunosenescence by boosting the immune system response to vaccination [6]. In Spain, three enhanced quadrivalent influenza vaccines (QIVs) are available, two of which are specifically licensed for persons aged ≥65 years [8,9,10]. Adjuvanted QIV (aQIV) contains a standard 15 μg dose of hemagglutinin plus a certain quantity of neuraminidase for each of the four influenza strains—A(H1N1), A(H3N2), B/Victoria, and B/Yamagata—included in the vaccine plus the MF59^®^ adjuvant, an oil-in-water emulsion of squalene oil that produces a stronger, broader, and longer immune responses relative to standard influenza vaccines in older adults [11,12]. High-dose QIV (HD-QIV) contains 60 μg of hemagglutinin for each of the four selected influenza viruses selected for the vaccine, which also produces a stronger immune response than standard influenza vaccines in older persons [13]. In addition, a recombinant QIV (QIVr) was recently licensed for adults ≥18 years of age. This vaccine contains 45 μg of hemagglutinin for each of the four vaccine virus strains and does not contain neuraminidase [9].

A recent modeling study suggested that QIVr might prevent more influenza cases, with associated reductions in influenza hospitalizations and deaths, than aQIV [14]. However, to date, no randomized, head-to-head clinical trials have compared the efficacy and no observational studies have compared the effectiveness of aQIV and QIVr. One observational study compared QIVr to the adjuvanted trivalent influenza vaccine (aTIV), the high-dose trivalent influenza vaccine (HD-TIV), and the egg-based QIV in older adults aged ≥65 years during the 2019–2020 season. These authors found that the relative vaccine effectiveness (rVE) of QIVr vs. aTIV was 5.6% (95% CI, −0.6 to 11.4) for the prevention of influenza-related hospital encounters and 10.7% (95% CI, 2.7–17.9%) for the prevention of influenza-related inpatient stays [15]. None of these studies have addressed cost effectiveness—an important factor in determining the overall value of a new vaccine to a community that helps inform decision-making by policy makers and healthcare providers. Using a modeling methodology established in previous studies [16,17,18,19,20], we recently assessed the cost effectiveness of aQIV and HD-QIV and found that aQIV provided cost savings relative to HD-QIV [21]. Here, we report the results of a similar modeling analysis conducted to evaluate the cost effectiveness of QIVr relative to aQIV.

## 2. Materials and Methods

### 2.1. Model Structure

**Figure 1:**
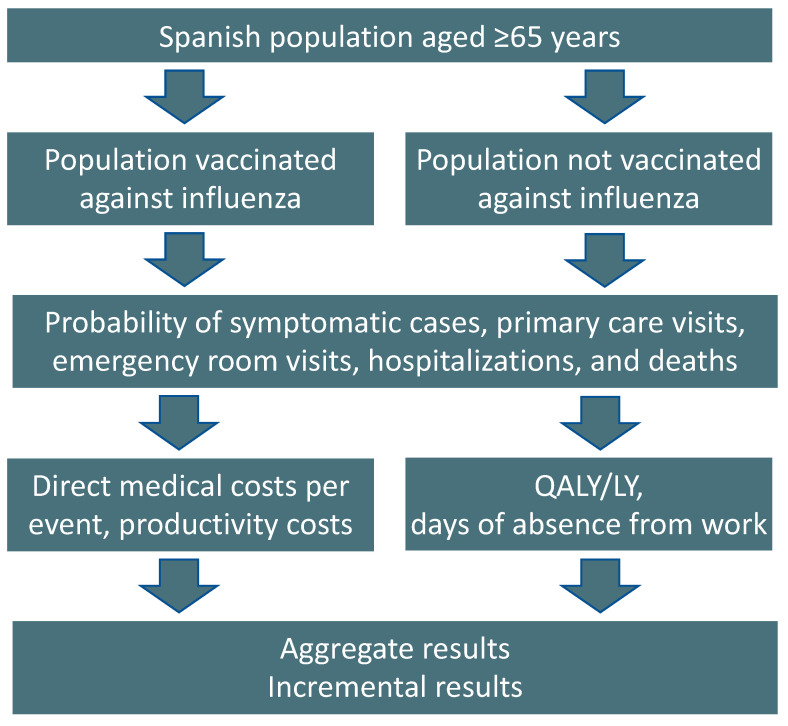
Schematic of the health economic model. LY, life year; QALY, quality-adjusted life year.

Similar to previously published studies, we used a health economic model to simulate the costs, benefits, and burden of disease for older adults aged ≥65 years in Spain vaccinated with either aQIV or QIVr during one influenza season (Figure 1). This model was based on a previously developed static, decision-tree model specific to the Spanish setting and designed in line with Spanish best practices for health economic modeling [21,22,23]. Several influenza cost-effectiveness analyses conducted in other countries have used a similar model [16,17,18,19,20].

### 2.2. Model Inputs and Calculations

The model was used to calculate the rates of different clinical outcomes per 100,000 subjects within a 1-year time horizon (representing one influenza season) among Spanish adults aged ≥65 years. The model included an average of clinically reported influenza cases in the Spanish population over three influenza seasons (2017–2020), as reported by the Sistema Centinela de Vigilancia de Gripe en España (ScVGE) (Table 1) [3].

In addition, the model used the inputs shown in Table 2. In the model, vaccinated individuals were assigned to either aQIV or QIVr. Over the 1-year time horizon and based on the average influenza incidence shown in Table 1, the model population was assigned to the following states: uninfected or asymptomatic; symptomatic cases not seeking medical support; or symptomatic cases requiring either a primary care visit, emergency department visit, or hospitalization. Hospitalized patients were further assigned a probability of death. Averted direct medical costs and indirect, societal costs were included in the model as deductions from incremental vaccine cost. Reduced disutilities during illness were included as additions to QALY (Table 2). Costs and outcomes calculations were for a full influenza season, except for productivity loss due to death and quality-adjusted life year (QALY) loss due to death. These costs were calculated over a lifetime horizon and discounted at 3% per year, following Spanish cost-effectiveness guidelines [23]. The 3% discount was applied to both costs and QALYs.

As shown in Table 2, the baseline utility (a health-related quality of life [HRQoL] input) for persons ≥65 years of age was estimated as 0.65 based on a German study of health and economic outcomes related to QIV vaccination. Data from the same study were used to estimate the disutility for symptomatic cases—0.32 for 7 days [37]. Disutilities for medically treated patients were derived from a longitudinal study of Spanish patients from major hospitals and primary care centers [38]. The disutility was calculated as the difference between patient-reported EQ-5D-3L score for the week prior to an influenza infection and the EQ-5D03L score during the infection. Calculated disutilities were 0.6 for inpatients and 0.33 for outpatients, with estimated durations of 21 and 7 days, respectively.

All costs were in 2021 euros and did not include administration or transportation costs, which are expected to be the same across vaccines. Tender prices for vaccines included in the model were EUR 13 for aQIV and EUR 25 for QIVr [27]. Resource unit costs for primary care, emergency department (ED), and hospitalizations were collected from official Spanish sources, including three bulletins of the Autonomous Communities: Andalucía, Murcia, and País Vasco [28,29,30,31]. The median values of the costs for primary care (EUR 59) and ED visits (EUR 183) were used in the model [28,29,30]. The weighted average cost of hospitalizations was calculated for influenza-related complications based on 2019 APR-DRG statistical data published by the Spanish Ministry of Health and inflated to the 2021 euro. These inputs included a 9-day intensive care unit stay for 7.5% of admissions, at a cost of EUR 4467 [30,31]. Individuals with symptomatic disease who did not have a primary care or ED visit or hospital admission were conservatively assumed to have no public payer or societal costs. A cost of EUR 25,000 per QALY gained was used as the Spanish willingness-to-pay threshold based on local pharmacoeconomics practices [40,41].

Lost productivity due to influenza infection was calculated using the discounted human capital approach and based on working days lost multiplied by the probability of being employed, or 1.2% for persons aged 65–69 years and 0.3% for those aged ≥70 years [33,42]. Productivity losses were assumed to be 5 working days for outpatients and 15 working days for inpatients, at an hourly rate of EUR 17.44 [22,34]. Productivity loss also included time spent by caregivers caring for influenza patients aged 65–69 years (5.4% of whom live with family members) and aged ≥70 years (14% of whom live with family) [35].

### 2.3. Vaccine Effectiveness Estimates

As mentioned, randomized, head-to-head clinical trials comparing the efficacy of aQIV and QIVr have not been conducted, and no observational studies have yet compared aQIV and QIVr. The only relevant comparison is a single observational study by Izurieta et al. comparing QIVr with an aTIV, as well as other formulations, in older adults aged ≥65 years during the 2019–2020 season. In this study, rVE was calculated as (1—relative risk [RR]) × 100%, and the rVE for the prevention of influenza-related inpatient stays was 10.7% (95% CI, 2.7–17.9%) [15]. This was the value used in our analyses.

### 2.4. Analysis

Two analysis scenarios were conducted to test the impact of the model assumptions on the incremental cost-effectiveness ratios (ICERs). One-way deterministic sensitivity analysis (DSA) was used to evaluate the impact of input uncertainty.

In the first analysis scenario, the rVE of 10.7% for QIVr vs. aQIV was used based on the QIVr vs. aTIV comparison reported in Izurieta et al. This value was used because it is the highest rVE available for a recombinant vs. and adjuvanted influenza vaccine [15]. Because the first scenario used an rVE calculated from a single influenza season, a second scenario was conducted. In this scenario, an incremental cost-effectiveness ratio of EUR 25,000 per QALY gained was used to determine the minimum rVE requirement for QIVr to be cost effective. To confirm the robustness of the analyses, a probabilistic sensitivity analysis (PSA) of the first scenario was conducted with 10,000 iterations of the model.

## 3. Results

In the first scenario based on a 10.7% rVE favoring QIVr over aQIV, QIVr was associated with incremental costs of EUR 71,054,601.74 and 699.27 QALY gained, for an ICER of EUR 101,612.41 per QALY gained. In the second scenario, to achieve a cost-effective ICER of EUR 25,000 per QALY gained, QIVr would need to be associated with an rVE of 34.12%, incremental costs of EUR 55,747,194.30, and QALY gained of 2229.88 (Table 3).

**Figure 2:**
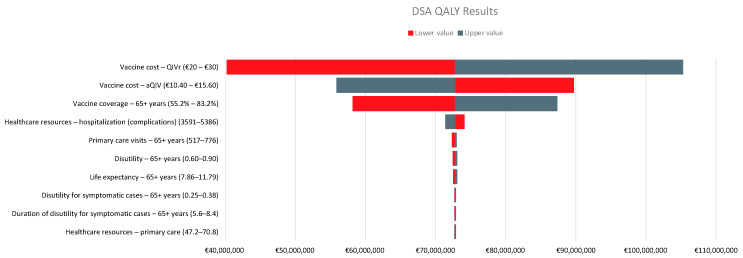
Tornado diagram showing the incremental net monetary benefit for recombinant quadrivalent influenza vaccine (QIVr) vs. adjuvanted quadrivalent influenza vaccine (aQIV) at a willingness-to-pay threshold of EUR 25,000 per quality-adjusted life year (QALY).

A DSA was conducted with the tornado plot summarizing the most influential parameters for the ICER presented in Figure 2. Vaccine costs are the most influential parameters in the model, followed by vaccine coverage. Other inputs have a relatively low impact on the cost effectiveness.

**Figure 3:**
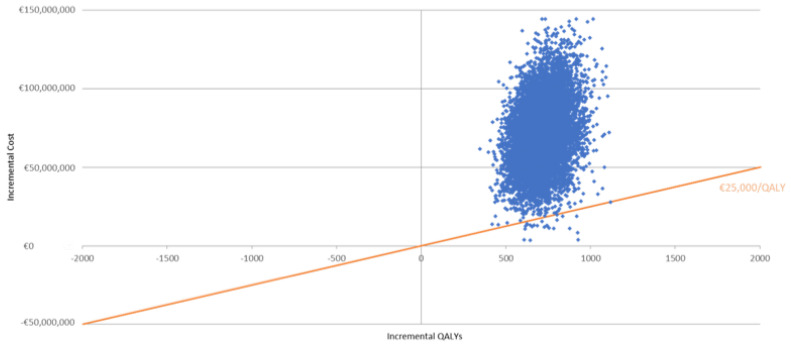
Cost-effectiveness plane for QIVr vs. aQIV in the probabilistic sensitivity analysis (PSA).

In the PSA conducted to confirm the robustness of the cost-effectiveness analysis, 99.7% of simulations were higher than the willingness-to-pay curve (Figure 3).

## 4. Discussion

Our results show that QIVr at its current price and the assumed increased effectiveness over aQIV is not cost effective for vaccination against influenza in older adults aged ≥65 years in Spain. At an ICER of EUR 101,612.41/QALY gained and at an rVE of 10.7% favoring QIVr (as considered in the model), the current tender price of EUR 25 per QIVr dose (vs. EUR 13 for aQIV [27]) is too high to be considered cost effective. PSA confirms the robustness of these findings. To meet the Spanish cost-effectiveness threshold of EUR 25,000/QALY gained, QIVr would need to be at least 34% more effective than aQIV, with an incremental cost of EUR 55,747,194.30 and an associated 2229.88 QALY gained. Such results would be difficult to achieve in clinical trials or in the real world. In the pivotal, randomized clinical trial comparing QIVr to nonadjuvanted, standard dose QIV, the vaccine efficacy of QIVr was 30% (95% CI, 10–47) vs. QIV in subjects ≥50 years of age but 17% (95% CI, –20 to 43) in adults aged >64 years [43]. Another way to increase the cost effectiveness of QIVr would be to lower the price. According to our model, if the tender price were less than EUR 16 per dose, then the cost effectiveness of QIVR would meet the presumed willingness-to-pay threshold of EUR 25,000 per QALY gained, using the available comparative effectiveness value of 10.7% from Izurieta et al., which is the only head-to-head study [15]. More studies should be conducted to establish a reliable rVE to estimate the price at which QIVr would be cost effective.

The rVE of 10.7% used in this analysis was based on a comparison of QIVr with an adjuvanted trivalent formulation, which was missing antigens for one of the B viruses contained within QIVr. No published studies have compared the efficacy or effectiveness of QIVr and aQIV, so it is unknown how inclusion of the fourth virus strain would affect the rVE value between these vaccines. QIVr contains three times the dose of hemagglutinin (45 μg per antigen) as standard dose vaccines, including aQIV (15 μg per antigen) and, thus, may be considered a “high-dose” influenza vaccine. The results of a meta-analysis of real world evidence comparisons between aTIV and HD-TIV (60 μg per antigen) showed no significant difference between the adjuvanted and high-dose formulations, with a point estimate of 3.2% (95% CI, –2.5 to 8.9), slightly favoring aTIV [44]. Based on these findings, it may be safe to presume that the rVE value of 10.7% favoring QIVr used in this modeling analysis was very conservative and that the actual difference in effectiveness between aQIV and QIVr would be small. If that were the case, the ICER for QIVr would be considerably higher.

Our group recently conducted another cost-effectiveness analysis comparing aQIV and HD-QIV in Spain, and our results in that study support the findings of the present analysis. In the previous study, we demonstrated cost savings of EUR 63.6 million if aQIV rather than HD-QIV were used to vaccinate a population of ~5 million older adults because aQIV is less expensive than HD-QIV and presents a minor advantage in effectiveness, with 5405 fewer symptomatic cases, 760 fewer primary care visits, 171 fewer emergency room visits, 442 fewer hospitalizations, and 26 fewer deaths, with a corresponding increase of 206 QALYs each year. In addition, aQIV fell below the Spanish cost-effectiveness threshold in this study [21]. The findings of both modeled comparisons—aQIV vs. HD-QIV and aQIV vs. QIVr—are consistent with cost-effectiveness studies of aQIV conducted in Europe and Latin America [45,46,47,48]. A study in Germany used a compartmental transmission model to predict the number of medically attended infections among adults ≥65 years vaccinated with aQIV or HD-QIV and assumed the rVE range of –2.5% to 8.9% for aQIV vs. HD-QIV, based on data from the meta-analysis of aTIV and HD-TIV comparisons [44,45]. In different modeling scenarios conducted based on influenza severity and the rVE range of –2.5% to 8.9% for aQIV vs. HD-QIV, aQIV was consistently shown to be cost saving because the unit cost of aQIV (EUR 19.21) was less than half that of HD-QIV (EUR 40.55), while vaccine effectiveness was similar. Even in the scenario where the rVE was –2.5% (favoring HD-QIV over aQIV), aQIV was more cost effective [45]. The same authors conducted a modeling study in the United Kingdom before HD-QIV was available there. The methodology included a similar clinical effectiveness of the two vaccines based on the meta-analysis cited above [44] and cost inputs based on a list price of GBP 11.88 for aQIV and the assumption that the HD-QIV price would be equivalent to the then-current price of HD-TIV of GBP 20.00. Similar to the German study, the authors showed that aQIV would be cost saving compared with HD-QIV unless the price of the high dose formulation range was no higher than GBP 12.94, similar to the current price of aQIV and considerably lower than the cost of HD-TIV [46]. In a 2015 study by our group in Spain, we performed a budget impact analysis that compared scenarios in which either aTIV or TIV were used to vaccinate adults older than 64 years, and numbers of influenza cases, medical consultations, pharmacological costs, and associated complications were evaluated. Costs were based on data from the Boletín Oficial de Andalucía for medical consultations and the Ministry of Health for influenza complications. The use of aTIV was associated with higher vaccination costs but these were offset by lower costs associated with reduced influenza cases and complications. Altogether, aTIV was associated with cost savings of EUR 76 million based on reductions in hospitalizations for influenza and its complications [47]. A modeling study conducted in Canada used an age-structured four-strain, dynamic transmission susceptible, exposed, infected, and recovered (SEIR) model along with data from Northern Hemisphere influenza seasons between 2012 and 2019 to evaluate the use of standard, egg-based QIV for individuals younger than 65 years and aTIV for individuals 65 years of age and older; egg-based QIV and HD-TIV for those younger than and those aged 65 and older, respectively; and cell-based QIV and aTIV for the respective age groups, with egg-based QIV for all ages as a baseline scenario. In this study, the cell-based QIV plus aTIV strategy was cost effective and the egg-based QIV plus aTIV strategy was cost saving, while the QIV plus HD-TIV strategy was not cost effective based on a higher list price but similar effectiveness as aTIV [48]. Taken together, these studies showing that adjuvanted influenza vaccines are more cost effective than high-dose influenza vaccines, which is priced higher than the adjuvanted vaccines but has similar effectiveness, lend further support to our conclusion that aQIV is more cost effective than QIVr at current prices in Spain. Although in our scenario, the effectiveness of QIVr was higher than that of aQIV, as discussed above, the difference was not substantial enough to offset the higher cost of the recombinant vaccine.

Our analysis is limited by the dearth of available data comparing adjuvanted with recombinant influenza vaccines. To date, only a single observational study from a single season has been published, which compared QIVr to aTIV rather than aQIV [15]. In addition, the rVE used in this study was based on inpatient stays, while we applied the value across all outcomes in our analysis (i.e., symptomatic cases, primary care visits, ED visits, hospitalizations, and deaths). As the quadrivalent formulations of these enhanced vaccines become more widely used and more outcomes data become available, firmer conclusions on the rVE and cost effectiveness of these options can be drawn. Another possible limitation is the 1-year time horizon for the model, which may not reflect the longer-term effectiveness of the vaccine or cases of cross-immunity. Longer-term, multi-season studies comparing aQIV, HD-QIV, and QIVr would help clarify whether there are differences between these vaccines’ abilities to confer cross-protection or long-term immunity through B cell activation, including the presence or absence of the adjuvant, differences in the hemagglutinin dose, and inclusion vs. exclusion of neuraminidase. Such studies would in turn further inform cost-effectiveness estimates for these vaccines. The model also does not account for herd immunity because it is static rather than dynamic. This conservative assumption, however, is unlikely to have an impact on the results, given that only a small proportion of the total Spanish population is vaccinated.

The model inputs used in the study were primarily drawn from Spanish data on outcomes such as influenza incidence, primary care and ED visits, hospitalizations, resource use, costs, vaccine coverage, and mortality. Although some utility data are from Spain, data from the UK, Belgium, and Germany were also used, as in previous Spanish influenza models [22].

## 5. Conclusions

Adults ≥65 years of age are most vulnerable to the impact of influenza, with the highest rates of infection and poor outcomes. Enhanced vaccines were developed to overcome the immunosenescence—an age-related process that leads to dysregulation of the innate and adaptive immune systems and less effective humoral and cellular immune responses against pathogens or to vaccines [6]—which is common in older persons. Adjuvanted and high-dose influenza vaccines provide older adults with maximal protection from influenza compared with standard-dose vaccines. The current costs of enhanced vaccines are higher than the cost of standard dose vaccines, but these costs may be offset by reductions in direct costs of medical care and indirect costs related to absenteeism and reduced productivity among not only the older adult population but also family members or other caregivers who miss work to care for sick older persons. The findings from the present analysis and another recently published analysis of aQIV vs. HD-QIV [21] support the use of aQIV as the currently most cost-effective enhanced vaccine for individuals ≥65 years in Spain.

## Figures and Tables

**Table 1:** Incidence rates of influenza-related clinical events per 100,000 subjects aged ≥65 years.

Event	2017–2018	2018–2019	2019–2020
Symptomatic cases	22,530	13,697	10,636
Primary care visits	950	545	445
ED visits	213	122	100
Hospitalizations	668	489	324
Deaths	40	29	19

ED, emergency department.

**Table 2:** Summary of parameters used as inputs for the model.

Parameter	Value	Reference
Percentage of population aged ≥65 years who were vaccinated	69.4%	[24]
Life expectancy for population aged ≥65 years	9.8 years	[25]
Size of population aged ≥65 years	9,371,743	[26]
aQIV tender price	EUR 13	[27]
QIVr tender price	EUR 25	[27]
Primary-care physician visit costs (per visit)	EUR 59	[28,29,30]
ED visit costs (per visit)	EUR 183	[28,29,30]
Hospitalization costs (per event)	EUR 4467	[30,31]
Co-medication costs (per influenza case)	EUR 3.21	[32]
Probability of being employed (65–69 years)	1.2%	[33]
Probability of being employed (≥75 years)	0.3%	[33]
Productivity loss per hour	EUR 17.44	[34]
Probability of requiring care at home (65–69 years)	5.4%	[35]
Probability of requiring care at home (≥75 years)	14%	[35]
Baseline utility for age ≥65 years	0.65	[36]
Disutility value for symptomatic patients	0.32	[37]
Disutility value for outpatient setting	0.33	[38]
Disutility value for inpatient setting	0.6	[38]
Disutility duration for symptomatic patients	7 days	[37]
Disutility duration in outpatient setting	7 days	[38]
Disutility duration in inpatient setting	21 days	[38]
Hospital mortality rate for population aged ≥65 years	6%	[39]
Discount rates for costs and outcomes *	3%	[23]

aQIV, adjuvanted quadrivalent influenza vaccine; ED, emergency department; QIVr, recombinant quadrivalent influenza vaccine. * Productivity loss due to death and quality-adjusted life year loss due to death are calculated over a lifetime horizon and discounted at 3% per year.

**Table 3:** Analyses of QIVr compared to adjuvanted influenza vaccine in adults aged ≥65 years.

Scenario	Current Tender Price	rVE	Incremental Cost	QALYs Gained	ICER
Izurieta 2021 rVE	EUR 25	10.70%	EUR 71,054,601.74	699.27	EUR 101,612.41
ICER = EUR 25,000/QALY gained	EUR 25	34.12%	EUR 55,747,194.30	2229.88	EUR 25,000.00

ICER, incremental cost effectiveness ratio; QALY, quality-adjusted life years; rVE, relative vaccine effectiveness.

## Data Availability

Not applicable.
